# Suicidality in primary care patients who present with sadness and anhedonia: a prospective European study

**DOI:** 10.1186/s12888-016-0775-z

**Published:** 2016-04-06

**Authors:** Berta Moreno-Küstner, Rebeca Jones, Igor Švab, Heidi Maaroos, Miguel Xavier, Mirjam Geerlings, Francisco Torres-González, Irwin Nazareth, Emma Motrico-Martínez, Carmen Montón-Franco, María José Gil-de-Gómez, Marta Sánchez-Celaya, Miguel Ángel Díaz-Barreiros, Catalina Vicens-Caldentey, Michael King

**Affiliations:** Department of Personality, Assessment and Psychological Treatment, University of Malaga (Maristan Network), Malaga, Spain; Division of Psychiatry, UCL, London, UK; Department of Family Medicine, University of Ljubljana, Ljubljana, Slovenia; Faculty of Medicine, University of Tartu, Tartu, Estonia; NOVA Medical School – UNLLisbon, Lisbon, Portugal; University Medical Center Utrecht, Utrecht, The Netherlands; CIBERSAM, University of Granada, Granada, Spain; Department of Primary Care and Population Health, University College London Medical School and Medical, Research Council General Practice Research Framework, London, UK; International University Loyola Andalucía, Seville, Spain; Centro de Salud Casablanca. (redIAPP, grupo Aragón) Departamento de Medicina y Psiquiatría, Universidad de Zaragoza, Zaragoza, Spain; Unidad Docente de Medicina Familiar y Comunitaria de La Rioja, La Rioja, Spain; Unidad Docente de Medicina Familiar y Comunitaria de Madrid, Coordinadora de Coordinadora de Direcciones de Continuidad Asistencial, Servicio Madrileño de Salud, Madrid, Spain; Centro de Salud de Vecindario, Gerencia de Atención Primaria de Gran Canaria, Las Palmas, Spain; Centro de Salud Son Serra, Unidad de Investigación de Atención Primaria de Baleares (redIAPP, grupo Baleares), Mallorca, Spain

**Keywords:** Anhedonia, Depression, Primary care, Suicidality, Cohort, Risk

## Abstract

**Background:**

Sadness and anhedonia (loss of interest in activities) are central symptoms of major depression. However, not all people with these symptoms meet diagnostic criteria for major depression. We aimed to assess the importance of suicidality in the outcomes for primary care patients who present with sadness and anhedonia.

**Method:**

Cohort study of 2,599 unselected primary care attenders in six European countries followed up at 6 and 12 months.

**Results:**

1) In patients with sadness and/or anhedonia who were not depressed at entry to the study, suicide plans (OR = 3.05; 95 % CI = 1.50–6.24; *p* = 0.0022) and suicide attempts (OR = 9.08; 95 % CI = 2.57–32.03; *p* = 0.0006) were significant predictors of developing new onset depression at 6 or 12 months. 2) In patients with sadness and/or anhedonia who met CIDI criteria for major depression at entry, suicidal ideation (OR = 2.93; 95 % CI = 1.70–5.07; *p* = 0.0001), suicide plans (OR = 3.70; 95 % CI = 2.08–6.57; *p* < 0.0001), and suicide attempts (OR = 3.33; 95 % CI = 1.47–7.54; *p* = 0.0040) were significant predictors of persistent depression at 6 or 12 months.

**Conclusions:**

Three questions on suicidality could help primary care professionals to assess such patients more closely without necessarily establishing whether they meet criteria for major depression.

## Background

Major depression is a relatively common disorder in primary care and is characterised by relapse and remission [1]. It can have serious consequences in terms of total burden of disease [2] and costs to society [3]. Premature deaths may be due to associated physical morbidity as well as death from unnatural causes such as suicide [4]. Suicidal ideation is a core symptom of major depression [5–7]. Sadness and loss of interest in activities (anhedonia) are central symptoms of major depression and thus questions about them are useful in screening people for the disorder. However, not all people with these symptoms meet diagnostic criteria for major depression. The depression section of the Composite International Diagnostic Interview (CIDI) begins with these two key questions and people who answer affirmatively to one or both continue with the interview to establish whether or not they meet criteria for the disorder [8]. Suicidal ideation is also a symptom of major depression^1^ but, like sadness and anhedonia, it may occur in the absence of a diagnosis of major depression. There is a lack of knowledge about depression outcomes in patients who screen positive for anhedonia and sadness, irrespective of whether or not they have a diagnosis of major depression. We aimed to study the outcomes of patients who are found to have sadness and anhedonia when they attend their family doctors. In particular, we examined whether the presence of suicidal ideation, plans and attempts had any predictive role in the onset or persistence of depressive disorder over 6 or 12 months follow-up.

## Method

### Study design

We analysed data from the Predict Europe (PE) and Predict Spain (PS) prospective cohorts, which were recruited to develop and validate a risk prediction algorithm for the onset of major depression in primary care attendees over 12 months. The PE and PS studies, which were conducted in six European countries, have been described in detail elsewhere [9–13]. They were approved by relevant ethical committees (The South East Multi-centre Research Ethics Committee) in each country.

### Setting

In the PE study, six European centres participated: 1) 25 general practices in the Medical Research Council’s General Practice Research Framework, distributed across the United Kingdom; 2) nine large primary care centres in Andalucía, Southern Spain; 3) 74 general practices distributed nationwide in Slovenia; 4) 23 general practices distributed nationwide in Estonia; 5) seven large general practice centres near Utrecht, The Netherlands; and 6) two large primary care centres in urban and rural areas of Portugal that include 25 general practitioners. In the PS study, seven provinces participated with 41 health centres and 231 physicians distributed throughout Spain: Malaga and Granada in southern Spain; Saragossa and La Rioja in northern Spain; Madrid, capital of Spain, situated in the centre; Las Palmas in the Canary Islands; and Majorca in the Balearic Islands. Each health centre covered a population of 15,000–30,000 inhabitants from a geographically defined area. The family physicians in each health centre work as a group, with extensive primary care teams.

### Participants

In the PE study, data were collected in consecutive attendees aged 18–75 years between April 2003 and September 2004. In PS, in the six Spanish provinces, the study population was recruited between October 2005 and February 2006. The seventh province, Malaga, recruited between October 2003 and February 2004 as it was already participating in the PE study. Exclusion criteria for all participant countries were an inability to understand the main language of the country, psychosis, dementia, and incapacitating physical illness. In the UK and The Netherlands, patients were recruited in health centre waiting rooms whereas in the other countries recruitment was conducted in discussion with the family physician.

A total of 11,299 people took part in both Predict cohorts [2, 6]. For this analysis we selected only persons who answered yes to one or both of the two screening questions (on anhedonia and sadness in the preceding six months) of the Depression Section of the Composite International Diagnostic Interview (CIDI) [14, 15]. A total of 7,985 (71 %) participants answered negatively to both of these CIDI screening questions and were excluded. A total of 3,314 patients answered positively to one or both and were included in this study. We excluded patients with no information about suicidal behavior (*n* = 27), diagnoses (*n* = 74) and those with no follow-up data at 6 and 12 months (*n* = 614).

### Measures

Our outcome variable was a DSM-IV^1^ (Diagnostic and Statistical Manual of Mental Disorders) diagnosis of major depressive disorder as assigned by the Depression Section of CIDI (Version 2.1) [14–16].

Our main exposure of interest was suicidality (comprising ideation, plans and attempts) in the preceding six months, which was also assessed by the CIDI using the following questions:

- o Did you feel so low you thought a lot about committing suicide? (ideation)
- o Did you make a plan as to how you might do it? (plan)
- o Did you attempt suicide? (attempt).

Other explanatory factors

The following baseline variables were considered to be potential confounders of the relationship between suicidal ideas, plans and attempts and major depression at follow up:

- Social and demographic factors: age, sex, civil status, educational level, living alone or with others and debt and financial strain [17].
- Physical and mental well-being, assessed by the 12-item Short Form (SF-12) [18, 19] and a question on the presence of long-standing illness, disability or infirmity.
- Alcohol misuse, assessed by Alcohol Use Disorders Identification Test (AUDIT) [20–22].
- Life-time screen for depression: we used the first two questions of the CIDI [14, 15, 21] again but this time posed with regard to during their lifetime.
- Anxiety symptoms using the anxiety section of the Primary Care Evaluation of Mental Disorders (PRIME-MD) [23, 24].
- Brief questions on the quality of sexual and emotional relationships with a partner, adapted from a standardized questionnaire [25].
- Childhood experiences of physical, emotional or sexual abuse [26].
- Presence of serious physical, psychological or substance misuse problems, or any serious disability, in persons who were close friends or relations of participants.
- Difficulties in getting on with people and maintaining close relationships were assessed using questions from a social functioning scale [27].
- Family history of serious mental disorder and/or suicide in first-degree family members [28].
- Recent life-threatening events, using a brief validated checklist [29].
- Experiences of discrimination on the grounds of sex, age, ethnicity, appearance, disability or sexual orientation using questions from a European study [30].
- Adequacy, availability and sources of social support from family and friends [31].

### Statistical methods

The outcome was major depression at 6 and 12 months follow-up. The main exposure variable was a four category measure of suicidality in the previous 6 months: no suicidal behaviour (reference category); suicidal ideation only; suicide plans; suicide attempts. We performed the statistical analysis separately in non-depressed individuals at baseline – group 1 (for whom a diagnosis of major depression at either 6 or 12 months follow up indicated onset) and in depressed people at baseline—group 2 (for whom a diagnosis of depression at follow up indicated a lack of recovery). We analysed measures of depression from both 6 and 12 months follow-up in a single mixed effects logistic regression model, accounting for correlations between repeated measures over time by specifying a random effect of individual. This method permitted us to include in the analysis individuals with incomplete data at follow-up. In addition, estimating the average odds of depression over the two time points may provide more reliable estimates of depression at follow-up. This was particularly the case among non-depressed people at baseline, where low numbers with a diagnosis of depression at follow-up and low numbers reporting any suicidality at baseline could otherwise result in unreliable and imprecise estimates.

In the first statistical model of the putative relationship between suicidal behaviour and onset or persistence of depression, we adjusted for age, sex, lifetime depression, and country. We then adjusted additionally in turn for each of 20 pre-specified potential confounding variables as detailed above. We accounted for correlations between patients within the same health centre by specifying a random effect of centre in all statistical models.

We performed sensitivity analyses to assess whether our results might be biased by missing follow up data using two different methods: (1) further adjustment for important predictors of missingness, and (2) inverse probability weighting for the probability of outcome data at follow up. Both methods make the assumption that follow up data are missing at random (MAR) conditional on the covariates included in the respective models.

## Results

Our final sample contained 2,599 patients who met CIDI criteria for sadness and/or anhedonia. We divided this sample into two cohorts. The first cohort (group 1) contained participants who were not depressed at baseline (*n* = 1,412). Ninety-four per cent (*n* = 1,326) had follow-up data at 6 months and 90 % (*n* = 1,265) at 12 months. The second cohort (group 2) consisted of participants who were depressed at baseline (*n* = 1,187), of whom 95 % (*n* = 1,124) had follow-up data at 6 months and 89 % (*n* = 1,051) at 12 months. Frequencies of all the sociodemographic variables assessed in our study are summarized in Table 1. Concerning the distribution of the suicidality, 82.6 % present no suicidal behaviour (*n* = 2147) while, 8.2 % had any suicidal ideation, 6.7 % plans and 2.5 % suicide attempts (Table 2).

**Table 1:** Demographic characteristics of the 2,599 participants in Predict Europe and Predict Spain with sadness and anhedonia

Demographic characteristics	No major depression at baseline		Major depression at baseline		Total	
	*N =* 1,412		*N =* 1,187		*N =* 2,599	
	N	%	N	%	N	%
Country						
United Kingdom	235	16.6	154	13.0	389	15.0
Spain	719	50.9	559	47.1	1,278	49.2
Slovenia	117	8.3	68	5.7	185	7.1
Portugal	110	7.8	152	12.8	262	10.1
The Netherlands	113	8.0	118	9.9	231	8.9
Estonia	118	8.4	136	11.5	254	9.8
Age mean (SD)	49.5 (14.9)		45.1 (14.0)		47.5 (14.7)	
Sex						
Female	1,080	76.5	934	78.7	2,014	77.5
Male	332	23.5	253	21.3	585	22.5
Marital status						
Married/living with partner	944	66.9	738	62.2	1,682	64.7
Separated ordivorced	119	8.4	145	12.2	264	10.2
Single	218	15.4	234	19.7	452	15.4
Widowed	131	9.3	70	5.9	201	7.7
Level of education						
Higher professional, nursing	287	20.3	291	24.5	578	22.2
Secondary education and trade	475	33.6	406	34.2	881	33.9
Primary or no education	650	46.0	490	41.3	1,140	43.9
Household status						
Not living alone	1,219	86.3	1,043	87.9	2,262	87.0
Living alone	193	13.7	144	12.1	337	13.0
Financial strain						
Comfortable	1,014	71.8	746	62.8	1,760	67.7
Difficulties	398	28.2	441	37.2	839	32.3

**Table 2:** Suicidality of the 2,599 participants in Predict Europe and Predict Spain with sadness and anhedonia

Suicidality	No major depression at baseline		Major depression at baseline		Total	
	*N =* 1,412		*N =* 1,187		*N =* 2,599	
	N	%	N	%	N	%
No suicidality	1230	87.1	917	77.2	2147	82.6
Suicidal ideation	93	6.6	121	10.2	214	8.2
Suicide plan	70	5.0	104	8.8	174	6.7
Suicide attemps	19	1.3	45	3.8	64	2.5

### Onset of depression in patients who were not depressed at baseline

**Fig. 1:**
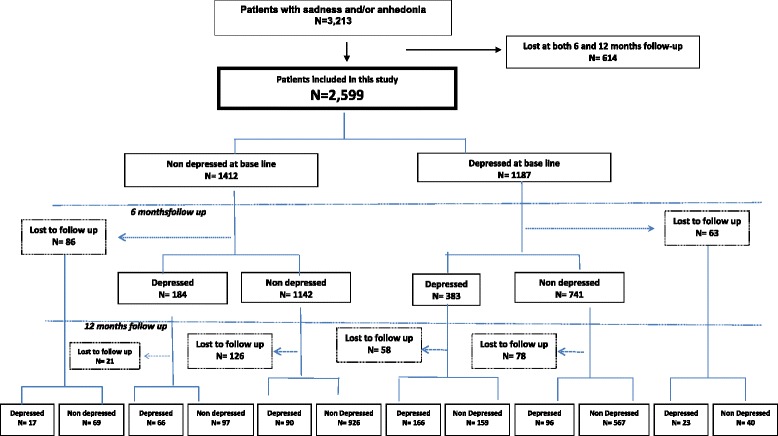
Flow diagram of recruitment and follow-up

Of the 1,412 participants who were not depressed at baseline and attended one or both of the follow up assessments, 13.9 % (184/1,326) received a diagnosis of major depression at 6 months, and 13.7 % (173/1,265) were depressed at 12 months (Fig. 1). In a model adjusted for age, sex, lifetime depression and country, suicidal ideation in the last 6 months was not a significant predictor of developing new onset depression at 6 or 12 months. However suicide plans in the last 6 months were a significant predictor (OR = 3.05; 95 % CI = 1.50 to 6.24; *p* = 0.0022), as was a history of suicide attempts in the last 6 months (OR = 9.08; 95 % CI = 2.57 to 32.03; *p* = 0.0006) (Table 2). There was no significant interaction between suicidal ideation and sex, or suicidal ideation and a three category measure of age.

### Persistent depression in patients who were depressed at baseline

Of the 1,187individuals, who were depressed at baseline and who attended one or both of the follow up assessments, 34.1 % (383/1,124) had persistent depression at 6 months, and 27.1 % (285/1,051) at 12 months (Fig. 1). In a similar multivariable model adjusted for age, sex, lifetime depression and country, suicidal ideation in the last 6 months was a significant predictor of persistent depression at 6 or 12 months (OR = 2.93; 95 % CI = 1.70 to 5.07; *p* = 0.0001),as were suicide plans in the last 6 months (OR = 3.70; 95 % CI = 2.08 to 6.57; *p* < 0.0001), and suicide attempts (OR = 3.33; 95 % CI = 1.47 to 7.54; *p* = 0.0040) (Table 2). Again, we found no significant interaction effects between suicidal ideation and sex, or suicidal ideation and a three category measure of age.

### Models with further adjustment

We found evidence of some degree of confounding in the relationship between suicidality and depression at 6 and 12 months by a number of pre-specified potential confounders, namely financial problems, panic attack, physical or emotional child abuse, sexual abuse, discrimination, social support and mental and physical quality of life (Table 3). Adjustment for a further 12 potential confounders did not materially alter the magnitude of the association, that is they did not affect the odds ratios by more than 5 % (results not shown).

**Table 3:** Association between suicidality (ideation, plans and attempts) and major depression at 6 or 12 months follow up in general practice patients with sadness and/or anhedonia

	Onset of depression (in non depressed people at baseline)			Persistent depression (in depressed people at baseline)		
	Suicidal ideation	Suicide plans	Suicide attempts	Suicidal ideation	Suicide plans	Suicide attempts
	OR (95 % CI) *p* value	OR (95 % CI) *p* value	OR (95 % CI) *p* value	OR (95 % CI) *p* value	OR (95 % CI) *p* value	OR (95 % CI) *p* value
Basic model						
Adjusted for age, sex, lifetime depression and country	1.17 (0.58 to 2.37) 0.6561	3.05 (1.50 to 6.24) 0.0022	9.08 (2.57 to 32.03) 0.0006	2.93 (1.70 to 5.07) 0.0001	3.70 (2.08 to 6.57) < 0.0001	3.33 (1.47 to 7.54) 0.0040
Further adjusted models						
Financial problems	1.09 (0.54 to 2.21) 0.7997	2.79 (1.37 to 5.68) 0.0045	8.49 (2.44 to 29.57) 0.0008	2.55 (1.48 to 4.41) 0.0008	3.55 (2.01 to 6.28) < 0.0001	3.05 (1.35 to 6.85) 0.0071
Panic attack	1.02 (0.50 to 2.08) 0.9653	2.80 (1.37 to 5.72) 0.0047	8.05 (2.28 to 28.46) 0.0012	2.62 (1.53 to 4.51) 0.0005	3.35 (1.90 to 5.90) < 0.0001	2.85 (1.28 to 6.35) 0.0106
Physical or emotional child abuse	1.07 (0.52 to 2.17) 0.8606	2.86 (1.40 to 5.82) 0.0039	8.54 (2.42 to 30.08) 0.0008	2.74 (1.59 to 4.72) 0.0003	3.28 (1.86 to 5.81) < 0.0001	2.95 (1.31 to 6.67) 0.0091
Sexual child abuse	1.13 (0.55 to 2.28) 0.7442	3.00 (1.47 to 6.14) 0.0026	8.95 (2.54 to 31.57) 0.0007	3.00 (1.74 to 5.16) 0.0001	3.33 (1.88 to 5.91) < 0.0001	3.07 (1.36 to 6.92) 0.0068
Discrimination	1.18 (0.58 to 2.37) 0.6509	2.88 (1.41 to 5.85) 0.0035	8.50 (2.41 to 29.98) 0.0009	2.72 (1.59 to 4.66) 0.0003	3.49 (1.98 to 6.12) < 0.0001	2.78 (1.24 to 6.24) 0.0131
Social support from family and friends	1.07 (0.53 to 2.15) 0.8561	2.85 (1.41 to 5.79) 0.0037	8.07 (2.29 to 28.39) 0.0011	2.67 (1.55 to 4.59) 0.0004	3.28 (1.86 to 5.79) < 0.0001	2.97 (1.32 to 6.69) 0.0088
Physical quality of life (SF12)	1.07 (0.53 to 2.15) 0.8516	2.90 (1.43 to 5.87) 0.0031	7.40 (2.16 to 25.35) 0.0014	2.75 (1.61 to 4.70) 0.0002	3.31 (1.88 to 5.82) < 0.0001	2.87 (1.29 to 6.40) 0.0099
Mental quality of life (SF12)	0.92 (0.46 to 1.85) 0.8075	2.33 (1.16 to 4.68) 0.0173	6.42 (1.89 to 21.77) 0.0028	2.53 (1.48 to 4.33) 0.0007	3.01 (1.72 to 5.28) 0.0001	2.62 (1.18 to 5.79) 0.0176

Notes: All statistical models adjusted for age, sex, lifetime depression and country. Further adjusted models adjusted additionally for each specified confounder in turn. All analyses accounted for correlations between patients within the same health centre through inclusion in statistical models of a random effect of centre and for correlations between repeated measures within patients through inclusion of a random effect of individual

The most important confounder was mental quality of life (SF12 mental health scale), which reduced the magnitude of the ORs for onset of depression in individuals who were not depressed at baseline (by 24 % for patients with suicide plans and 29 % for those with suicide attempts, relative to patients with no suicidality). However, even in this most extreme case, there was still evidence of a strong association between suicidality and the onset of depression, and adjusted ORs remained relatively large in magnitude (OR for onset in individuals with suicide plans: 2.33; 95 % CI: 1.16 to 4.68; OR for onset in individuals with a history of suicide attempts: 6.42; 95 % CI: 1.89 to 21.77).

In patients who were depressed at baseline, the most important confounder was again mental quality of life (SF12 mental health scale), which reduced the magnitude of the ORs (by 14 % for patients with suicidal ideation, 19 % for those with suicide plans and 21 % for those with suicide attempts, relative to patients with no suicidal behaviour). There was likewise still evidence of a strong association between suicidality and persistent depression, and adjusted ORs again remained relatively large in magnitude (OR for persistent depression in individuals with suicidal ideation: 2.53: 95 % CI: 1.48 to 4.33; OR for persistent depression in individuals with suicide plans: 3.01; 95 % CI: 1.72 to 5.28; OR for persistent depression in individuals with a history of suicide attempts: 2.62; 95 % CI: 1.18 to 5.79).

### Sensitivity analysis

The effect estimates from the further adjusted model (for important predictors of missingness) were very close to the results from the original model, while those from the inverse probability weighting analysis suggested that the original model may have slightly overestimated the association between suicidality and onset of depression and slightly underestimated the association between suicidality and persistent depression (Table 4). However, the results were not substantially different to those from the original analyses in either case, and would not result in any alteration to the conclusions drawn from the initial analysis.

**Table 4:** Comparison of results from original analysis with results from sensitivity analyses to assess the possibility of bias due to missing outcome data at follow up using two different methods

	Onset of depression (in non-depressed people at baseline)			Persistent depression (in depressed people at baseline)		
	Suicidal ideation	Suicide plans	Suicide attempts	Suicidal ideation	Suicide plans	Suicide attempts
	OR (95 % CI) *p* value	OR (95 % CI) *p* value	OR (95 % CI) *p* value	OR (95 % CI) *p* value	OR (95 % CI) *p* value	OR (95 % CI) *p* value
Basic model						
Adjusted for age, sex, lifetime depression and country	1.17 (0.58 to 2.37) 0.6561	3.05 (1.50 to 6.24) 0.0022	9.08 (2.57 to 32.03) 0.0006	2.93 (1.70 to 5.07) 0.0001	3.70 (2.08 to 6.57) < 0.0001	3.33 (1.47 to 7.54) 0.0040
Sensitivity analyses						
Basic model further adjusted for important predictors of missing outcome data at follow up(educational level and alcohol dependency)	1.13 (0.56 to 2.30) 0.7278	2.93 (1.43 to 6.01) 0.0033	8.53 (2.41 to 30.15) 0.0009	2.95 (1.70 to 5.11) 0.0001	3.60 (2.02 to 6.41) < 0.0001	3.15 (1.38 to 7.16) 0.0063
Basic model weighted by inverse of probability of outcome data at follow up	1.10 (0.61 to 1.99) 0.7533	2.69 (1.40 to 5.16) 0.0029	7.50 (1.90 to 29.65) 0.0041	3.44 (1.74 to 6.79) 0.0004	3.87 (2.34 to 6.39) < 0.0001	3.39 (1.29 to 8.94) 0.0134

Notes: All statistical models adjusted for age, sex, lifetime depression and country. Further adjusted models adjusted additionally for each specified confounder in turn. All analyses accounted for correlations between patients within the same health centre through inclusion in statistical models of a random effect of centre and for correlations between repeated measures within patients through inclusion of a random effect of individual

## Discussion

To our knowledge, this is the first study of the natural history of people experiencing sadness and anhedonia who attended primary medical care services and, in particular, of the role of suicidality in the subsequent onset or persistence of major depression. Most information concerning suicidal behaviour and major depression stems from studies of the general population or psychiatric services [32–34]. Considerably less evidence comes from primary care populations [35].

Our basic model (adjusted for age, sex, country and lifetime depression) showed that suicide plans and attempts in the preceding 6 months were significant predictors of both the onset and persistence of major depression in people presenting with sadness and/or anhedonia over 6 and 12 months of follow up, while ideation was a significant predictor only of persistence of depression. Although these results were attenuated after adjustment for a further set of potential confounders, the findings remained essentially the same and accord with those of Oquendo et al. [36], who conclude that suicidal behaviour is an important risk factor for major depression.

### Characteristics of suicidality

While suicidal ideation were not associated with the onset of major depression in this vulnerable group of people experiencing sadness and anhedonia, suicide plans and attempts increased the odds of onset of depression three- and nine-fold respectively. In those who were already depressed at recruitment, both suicidal ideation and attempts were associated with persistent depression, but the strongest risk factor was suicide plans.

This range of self-harming behaviours [37], starting with suicidal ideas, followed by making a plan, and finally attempting suicide [38], appears to function as a continuum of severity in relation to the onset of depression in people already predisposed by the experiences of sadness and anhedonia. However, in those attenders in whom sadness and anhedonia were part of a full syndrome of major depression, suicide plans appeared to have the strongest association with lack of recovery. Despite these differences, suicidality in its various forms is an essential part of the picture and suggests that primary care physicians should pay attention to it not only in their depressed patients but also in patients who seem to have a prodromal form of the disorder. Some years ago, Arroll et al. [39] suggested that two questions on feeling down or sad, and loss of interest in things were useful screening questions to use in general practice to identify attenders with possible depression. Our results indicate that for patients who answer affirmatively, one or two further questions on suicidality may well be useful in both identifying who is about to become depressed and who is likely to recover in those already depressed.

### Limitations of the study

One limitation in this study is the varying response to recruitment in each country, although the overall response was acceptable [9–11]. Furthermore, although personality disorders [40] are implicated in the onset of depression they were not included as potential risks in the development of the predict algorithm. This was because they are too complex to measure accurately in a brief prediction algorithm that was developed for use in family practice. We did not employ a specific instrument to measure suicidality but instead used questions which are part of the Depression Section of CIDI. A final limitation was the proportion of missing outcomes which might have biased our results; however, our sensitivity analysis showed that this was unlikely.

### Clinical implications and assessment of suicidality

People in countries where primary medical care is free at the point of access have ready access to general practitioners and develop long-term relationships with them [41]. Thus, primary care is an ideal setting in which to collect information for use in prevention. Once physicians establish that a patient has persistent sadness and/or anhedonia using two screening questions [39], three further questions on suicidal ideation, plans and attempts would help them to assess whether a patient 1) might be at risk of the onset of major depression or 2) if depressed, how likely they are to recover in the near future. Our data suggest that querying individuals on suicidal ideation would be clinically useful not only for persistent depression but also for the onset of depression. Family physicians do not always ask about suicide [42], despite recommendations that they should, especially in those with signs of depression [43, 44]. So this approach might provide a useful framework in which to do so.

## Conclusions

As our study demonstrates, patients may have suicidal inclinations even when they do not meet the full criteria for major depression. Three questions on suicidality could help primary care professionals assess patients who have persistent sadness and/or anhedonia more closely, without necessarily establishing whether they meet criteria for major depression. The need for these additional questions is supported by findings that only a minority of primary attendees tell their physicians about their thoughts and plans regarding suicide [45, 46]. Given that screening all patients in primary care practices for suicidal behaviour is neither practicable nor useful [47], the results of our study suggest that early detection of suicidal ideation, plans and attempts in persons with the key symptoms of sadness or anhedonia may enable the general practitioner to initiate timely interventions to prevent or ameliorate major depression.

## Consent

All patients gave fully informed consent for their data to be analysed and the results published.

### Availability of data and materials

Inquiries regarding the data should be addressed to the corresponding author.
